# Environmental perturbation of the circadian clock during pregnancy leads to transgenerational mood disorder-like behaviors in mice

**DOI:** 10.1038/s41598-017-13067-y

**Published:** 2017-10-03

**Authors:** Peng Zhang, Guang Li, Hui Li, XiaoQiu Tan, Hai-Ying Mary Cheng

**Affiliations:** 1 Key Laboratory of Medical Electrophysiology, Ministry of Education, Institute of Cardiovascular Medicine; Collaborative Innovation Center for Prevention and Treatment of Cardiovascular Disease of Sichuan Province; Southwest Medical University, Luzhou, 646009 China; 2 Department of Biology, University of Toronto Mississauga, 3359 Mississauga Road, Mississauga, ON L5L 1C6 Canada

## Abstract

It remains unknown whether chronic circadian disturbance (CCD) during pregnancy can lead to mood disorders in the offspring. Here we show that pregnant mice in the F0 generation that were exposed to CCD stress displayed depression-like behaviors, and produced offspring in the F1 and F2 generations that also exhibited mood-associated behavioral phenotypes despite the lack of direct stressful experiences during their postnatal or adult period. Prenatal CCD stress was correlated with the elevation of plasma corticosterone levels in F1 mice. Furthermore, the diurnal expression profiles of core circadian clock genes were disrupted in the suprachiasmatic nucleus of F1 mice. Proteomics analysis revealed that prenatal CCD stress resulted in distinct changes in protein expression in the hypothalamus of female F1 mice, in particular proteins that were associated with cellular activities, metabolism, development and diseases. Sex-specific differences in melanocortin 4 receptor expression were apparent in the CCD F1 generation. We conclude that maternal exposure to chronic circadian disturbance during pregnancy can lead to sex-specific mood disorders that persist for at least two filial generations. The underlying mechanisms may depend on transgenerational changes in plasma corticosterone levels, circadian pacemaking, and hypothalamic protein expression.

## Introduction

Mood disorders including depression and anxiety are serious conditions: according to the World Health Organization: more than 76.4 million people are afflicted by depression alone. Mood disorders can lead to reduced quality of life and risk of suicide, and are a serious socio-economic burden^1^. The causes of mood disorders are poorly understood, but a prevailing hypothesis is that stressful experiences in early life can lead to the development of mood disorders that may be transmitted to the next generation^2^. Many studies have shown that if a woman suffers from depression, anxiety or other stresses during pregnancy, her offspring is more vulnerable to developing emotional, behavioral and cognitive problems^3,4^. Prenatal stress can also lead to pregnancy-induced hypertension in the mother^5^, pre-term birth, lower birth weight, and altered physical outcomes for the offspring such as increased risk of asthma^6^. Animal studies have shown that prenatal stress can result in depression-like behavior and developmental and functional changes in the hypothalamic-pituitary-adrenal (HPA) axis of the offspring^7^. The HPA axis is highly sensitive to prenatal stress and may be programmed during fetal development^8^.

Some aspects of modern life such as rotating shift work impose stress on individuals by disrupting their circadian rhythms. Circadian rhythm disturbance has been implicated in a variety of psychiatric disorders including depression and anxiety^9^. Circadian rhythm disturbance is thought to underlie the core symptoms of major depression disorder (MDD), namely low mood and anhedonia^10^. Studies have linked shift work with adverse mental health outcomes in nurses, a key demographic that undertakes shift work^11–13^. It remains to be determined whether the offspring of women who perform shift work while pregnant are more susceptible to developing mental or mood disorders later in life.

To address this issue, we developed a chronic circadian disturbance (CCD) paradigm in mice, and found that CCD stress in pregnant females of the F0 generation leads to depression-like behaviors in the offspring (F1 generation) and in the offspring’s progeny (F2 generation). Diurnal oscillations of expression of the core circadian clock genes, *Clock*, *Bmal1*, *Per1* and *Per2*, in the suprachiasmatic nucleus (SCN) of F1 mice were perturbed by prenatal CCD stress. Quantitative proteomics using isobaric tags for relative and absolute quantitation (iTRAQ) revealed distinct changes in hypothalamic protein expression in CCD F1 females, with 57.4% and 19.7% of the differentially expressed proteins being involved in metabolic and developmental processes, respectively. Abundance of melanocortin 4 receptor (MC4-R), a key player in neuroendocrine processes, was markedly altered in the hypothalamus of the F1 generation. Overall, our study suggests that chronic disruption of circadian rhythms during pregnancy may lead to mood disorder-like behaviors in the offspring that may in part be due to perturbed circadian rhythms that persist into adulthood as well as neuroendocrine dysfunction.

## Results

### Gestational CCD stress induces mood disorder-like behaviors in the pregnant mice

**Figure 1:**
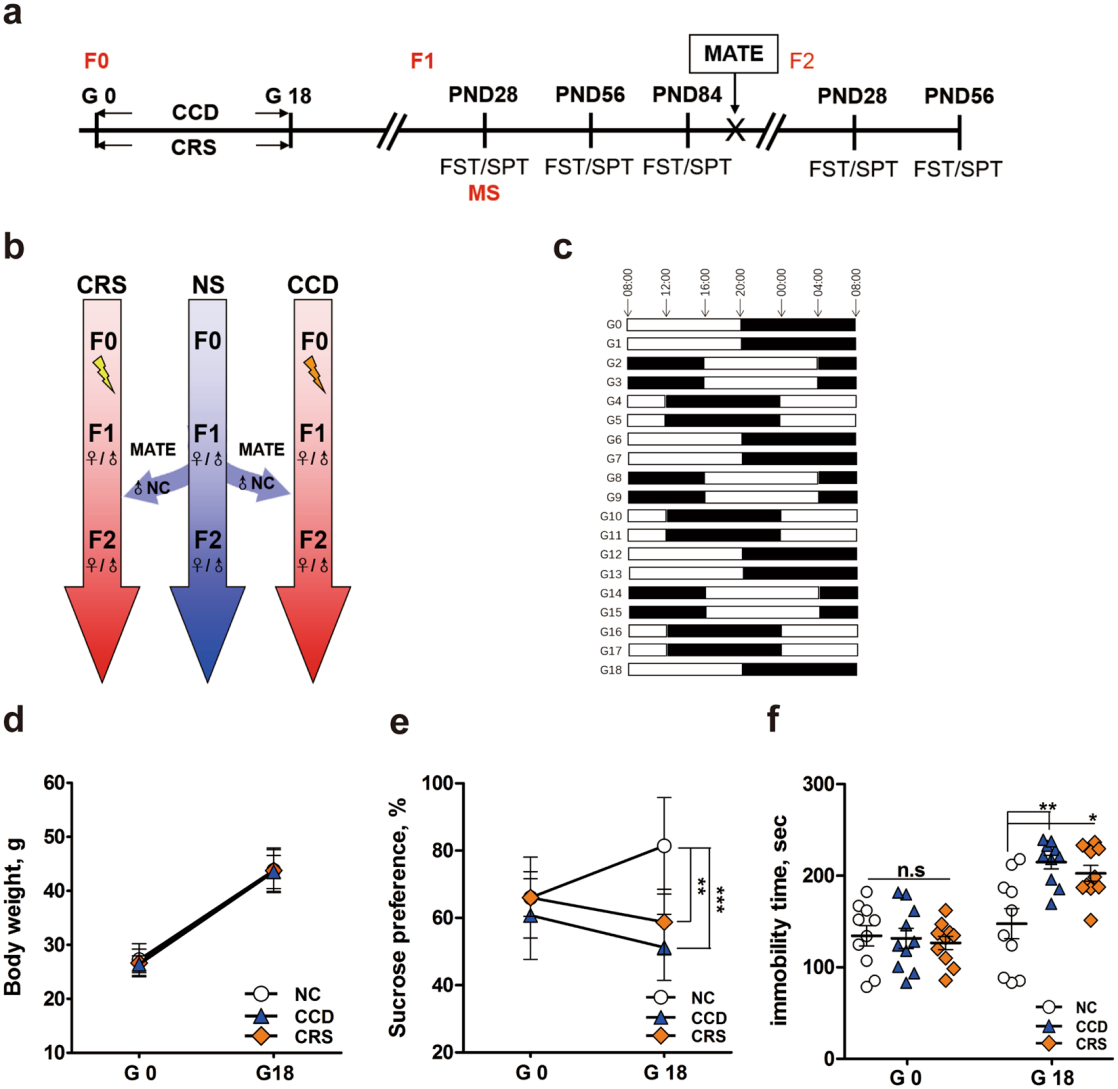
Experimental design and behavioral analyses of pregnant F0 mice in the CCD, CRS and NS groups. (**a**,**b**) Flow charts illustrate the experimental design, including the strategy and time course of animal breeding, stress exposures and behavioral tests in the three generations. (**c**) Depiction of the CCD paradigm, in which the LD cycle is delayed by 8 hours every two days from G0 to G18. (**d**–**f**) Body weight (**d**), sucrose preference (**e**) and immobility times in the forced swim test (**f**) of pregnant F0 mice at G0 and G18 in the CCD, CRS and NS groups. *P < 0.05, ***P < 0.001 vs. NS F0 group, n = 10 per group.

The breeding strategy and the two stress paradigms, chronic restraint stress (CRS) and CCD stress, are depicted in Fig. 1a,b. Prenatal CRS was used as a reference control, because it was previously reported to induce mood disorder-like behavior in the offspring^1,14,15^. Non-stressed (NS) pregnant female mice were used as negative controls. The CCD protocol involves 8-hour phase delays of the light-dark schedule every 2 days (Fig. 1c). Gestational CRS and CCD stress did not affect the body weight gain of pregnant F0 mice or induce spontaneous abortion (Fig. 1d and Table 1), suggesting that the stimulus intensities used in this study were modest. Plasma corticosterone levels in F0 mice at Zeitgeber time (ZT) 8 of gestation day 18 (G18) were measured. CCD and CRS F0 female mice had elevated corticosterone levels at G18 compared with NS controls (Fig. S1a), although the increase was more pronounced under CRS than it was under CCD stress. The difference in corticosterone levels between CCD and CRS F0 females may be due to a shift in the corticosterone rhythms under the CCD paradigm. The sucrose preference and forced swim tests were used to assess anhedonia- and depression-like behaviors, respectively. Relative to NS controls, sucrose preference was significantly reduced in pregnant F0 mice under CRS and CCD stress at G18 but not at G0 (Fig. 1e). At G18, CRS and CCD F0 females also exhibited prolonged immobility time in the forced swim test compared with NS controls (Fig. 1f). These results indicate that our CCD paradigm induces mood disorder-like behaviors in mice.

**Table 1:** Body weight of F0 mice and sex ratio of F1 mice.

	NS			CCD			CRS		
F0	Number	Body	SD	Number	Body	SD	Number	Body	SD
		weight, g			weight, g			weight, g	
		(mean)			(mean)			(mean)	
	10	27.27	1.96	10	26.41	1.57	10	26.67	1.55
		(G0)			(G0)			(G0)	
	10	43.8	2.12	10	43.5	3.05	10	43.77	2.88
		(G18)			(G18)			(G18)	
F1	litter size	Sex ratio	SD	litter size	Sex ratio	SD	litter size	Sex ratio	SD
	(mean + SD)	(♀: ♂)		(mean + SD)	(♀: ♂)		(mean + SD)	(♀: ♂)	
	10.2 + 1.23	1.12	0.39	9.8 + 1.55	1.75***	0.29	9.9 + 0.88	1.11	0.4

***P = 0.0006, vs. NS F1 group. Note: litter size and sex ratio were determined at the time of weaning.

### Prenatal CCD and CRS stresses cause differential phenotypes of mood disorder-like behaviors and HPA axis responses in the F1 generation

**Figure 2:**
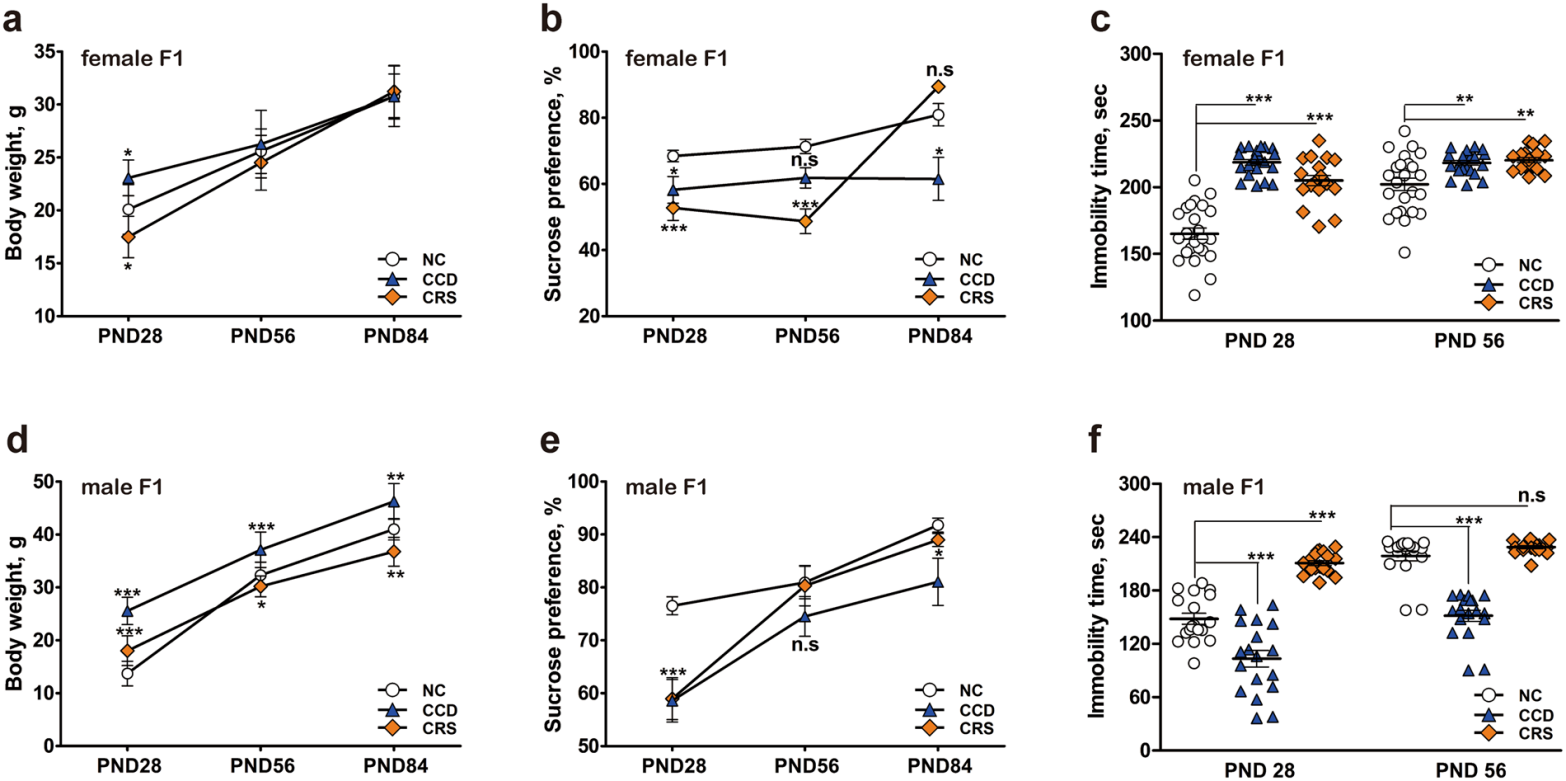
Effects of prenatal stresses on the body weight, sucrose preference and FST immobility times of F1 mice in the CCD, CRS and NS groups. (**a**,**d**) Body weight of female (**a**) and male (**d**) F1 mice at PND 28, 56 and 84 in the CCD, CRS and NS groups. (**b,e**) Sucrose preference of female (**b**) and male (**e**) F1 mice at PND 28, 56 and 84 in the CCD, CRS and NS groups. (**c**,**f**) FST immobility times of female (**c**) and male (**f**) F1 mice at PND 28, 56 in the CCD, CRS and NS groups. *P < 0.05, **P < 0.01, ***P < 0.001 vs. sex-matched NS F1 group, n = 33 female mice per group, n = 18 male mice per group.

Next, we examined the effects of prenatal CCD and CRS stress on the F1 generation. Sex ratios and litter sizes of the F1 generation were determined at the time of weaning (Table 1). Litter sizes were comparable between CCD, CRS and NS groups (Table 1), but the sex ratio was significantly skewed towards female for the CCD F1 group (Table 1). We noted that some of the CCD F0 dams cannibalized 2 or 3 pups within days of birth, although this was not observed for all 10 litters. This may account for the extreme skewing of the sex ratio in this treatment group. In addition, the body weight of 28-day-old mice was different when comparing the three treatment groups (Fig. 2a,d). CCD F1 mice of both sexes were significantly heavier than NS controls (Fig. 2a,d). By 56 days of age, the body weight of CCD F1 female mice was comparable to that of NS controls (Fig. 2a), whereas the difference in male body weight was apparent even at 84 days of age (Fig. 2d). The difference in F1 body weight at 28 days may reflect a potential effect of gestational CCD on nursing behavior of lactating dams. However, altered feeding behavior and metabolism of CCD F1 mice, particularly the males, may account for their greater body weight even at a more advanced age.

In the absence of stressors during postnatal life, F1 mice that had been exposed to CCD stress *in utero* displayed certain mood disorder-like behaviors. In the sucrose preference test, both female and male CCD F1 mice displayed signs of anhedonia as reflected by the reduction of sucrose preference at 28 and 84 days of age (Fig. 2b,e). Significant reductions in sucrose preference were also observed in the CRS group, specifically 28- and 56-day-old F1 females (Fig. 2b), and 28-day-old F1 males (Fig. 2e) relative to NS F1 controls. In the forced swim test, regardless of sex, F1 mice that were exposed to CRS *in utero* exhibited increased immobility time relative to NS controls (Fig. 2c,f). *In utero* exposure to CCD stress similarly prolonged immobility time in F1 female mice (Fig. 2c), but decreased it in F1 male mice (Fig. 2f). Altogether, these results indicate that prenatal CCD stress can result in anhedonia-like behavior in the adult offspring of both sexes, and depression-like behavior in the female offspring only.

In addition to behavioral tests, we measured plasma corticosterone levels in 28- and 56-day-old F1 mice at Zeitgeber time (ZT) 8. Prenatal CCD stress elevated corticosterone levels in both male and female F1 mice, although this increase was not sustained in females at 56 days of age (Fig. S1b and S1c). In contrast, prenatal CRS stress had minimal effect on plasma corticosterone levels in F1 mice, with the exception of an increase in 28-day-old F1 males (Fig. S1b and S1c). Collectively, these data indicate that prenatal CCD stress not only affects the mood of the offspring once they reach adulthood, but also their endocrine function.

### Prenatal CCD stress alters the diurnal expression pattern of core circadian clock genes in the SCN of the F1 generation

**Figure 3:**
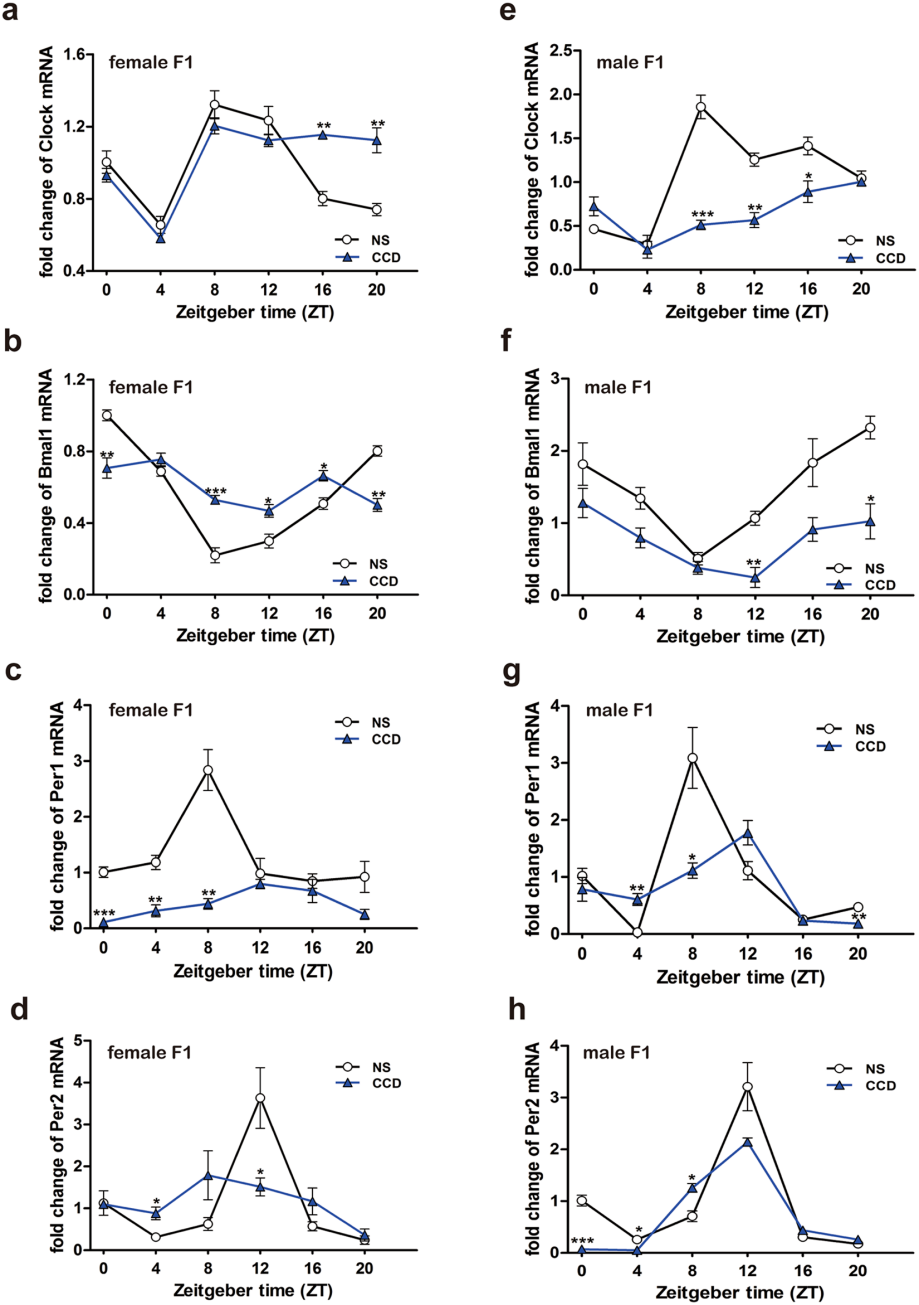
mRNA and protein expression patterns of Clock and Bmal1 in the SCN of F1 mice in the CCD, CRS and NS groups. (**a–h**) Diurnal oscillations of *Clock* (a, e), *Bmal1* (**b**,**f**), *Per1* (**c**,**g**) and *Per2* (**d**,**h**) transcripts in the SCN of NS F1 and CCD F1 mice, n = 3 per time point per group, n = 3 per group. *P < 0.05, **P < 0.01, ***P < 0.001 vs. NS F1 group.

Given that circadian rhythm disturbances have been implicated in mood disorders, we hypothesized that prenatal CCD stress may perturb circadian rhythms of the F1 generation, contributing to the observed mood disorder-like behaviors. In order to determine whether circadian rhythms are affected in the F1 animals, we employed quantitative reverse transcription PCR (RT-qPCR) to analyze the diurnal expression profiles of the core circadian clock genes, *Clock*, *Bmal1*, *Per1* and *Per2*, in the SCN of the anterior hypothalamus^16^, which serves as the master circadian pacemaker, over a 24-hour period (Fig. 3). In NS F1 mice, robust rhythms in transcript abundance were observed for these clock genes, with temporal profiles that were comparable between both sexes (Fig. 3a–h). Prenatal CCD stress altered the diurnal expression of these genes in a sex-specific manner (Fig. 3a–h). With the exception of *Clock* in female F1 mice (Fig. 3a), prenatal CCD stress reduced the oscillatory amplitude of circadian clock gene expression (Fig. 3b–h). In most cases, amplitude reduction was due to a decrease in transcript levels across the 24 h cycle (Fig. 3c–h). Prenatal CCD stress had a more marked effect on *Per1* and *Per2* expression in female compared to male mice (Fig. 3c,d,g and h), whereas the reverse was true for *Clock* and *Bmal1* expression (Fig. 3a,b,e and f). These data support the conclusion that prenatal CCD stress alters diurnal rhythms of clock gene expression in the SCN pacemaker of the F1 generation.

### Gestational CCD stress in the F0 generation perturbs mood-associated behaviors in the F2 generation

**Figure 4:**
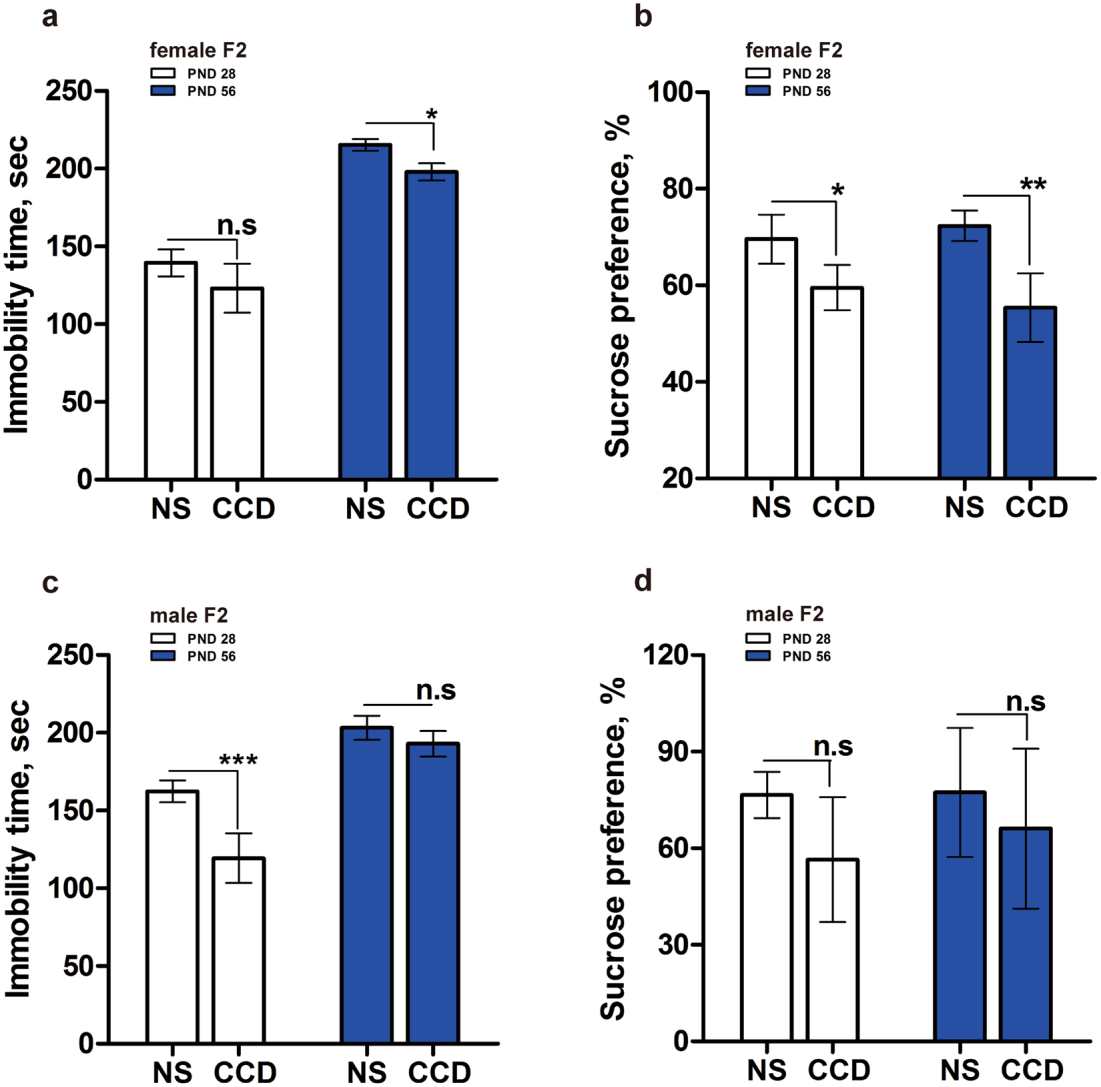
Gestational CCD stress in the F0 generation leads to mood disorder-like phenotypes in the F2 generation. (**a**,**c**) FST immobility times of female (**a**) and male (**c**) F2 mice at PND28 and 56. (**b**,**d**) Sucrose preference of female (**b**) and male (**d**) F2 mice at PND28 and 56. *P < 0.05, **P < 0.01, ***P < 0.001 vs. sex-matched NS F2 group, n = 15 per group.

To assess whether the behavioral consequences of gestational CCD stress extended beyond the F1 generation, we subjected female and male F2 mice to the sucrose preference and forced swim tests at 28 and 56 days of age. In the sucrose preference test, CCD F2 female mice showed a significant reduction in sucrose preference at both ages (Fig. 4b), paralleling the behavior of CCD F1 females. On the other hand, CCD F2 male mice were indistinguishable from NS controls in terms of sucrose preference (Fig. 4d), suggesting that the anhedonic effects of gestational CCD stress on F0 dams only persist to the F2 generation in females but not males. Sucrose preference was also modestly attenuated in CRS F2 female mice at 28 days of age and in CRS F2 male mice at 56 days of age (Fig. S3b,Sd).

Unexpectedly, unlike the F1 generation, F2 mice from both gestational stress paradigms exhibited reduced, not prolonged, immobility times in the force swim test in a sex- and age-dependent manner. Immobility time was significantly reduced in 56-day-old CCD F2 females (Fig. 4a), 28-day-old CCD F2 males (Fig. 4c), 28- and 56-day-old CRS F2 females (Fig. S3a), and 28-day-old CRS males (Fig. S3c) relative to NS controls. Although it is not clear why gestational CCD stress in the F0 generation leads to depressive behavior in F1 females, but anti-depressive behavior in F2 mice of both sexes, our results nevertheless indicate that mood-related behaviors are perturbed in the F2 generation. Altogether, our data suggest that gestational CCD stress in the F0 generation can impact mood-associated behaviors in the F2 generation, in particular anhedonia in females and anti-depressive behavior in both sexes.

### Gestational CCD stress alters the hypothalamic proteome of CCD F1 female mice

**Figure 5:**
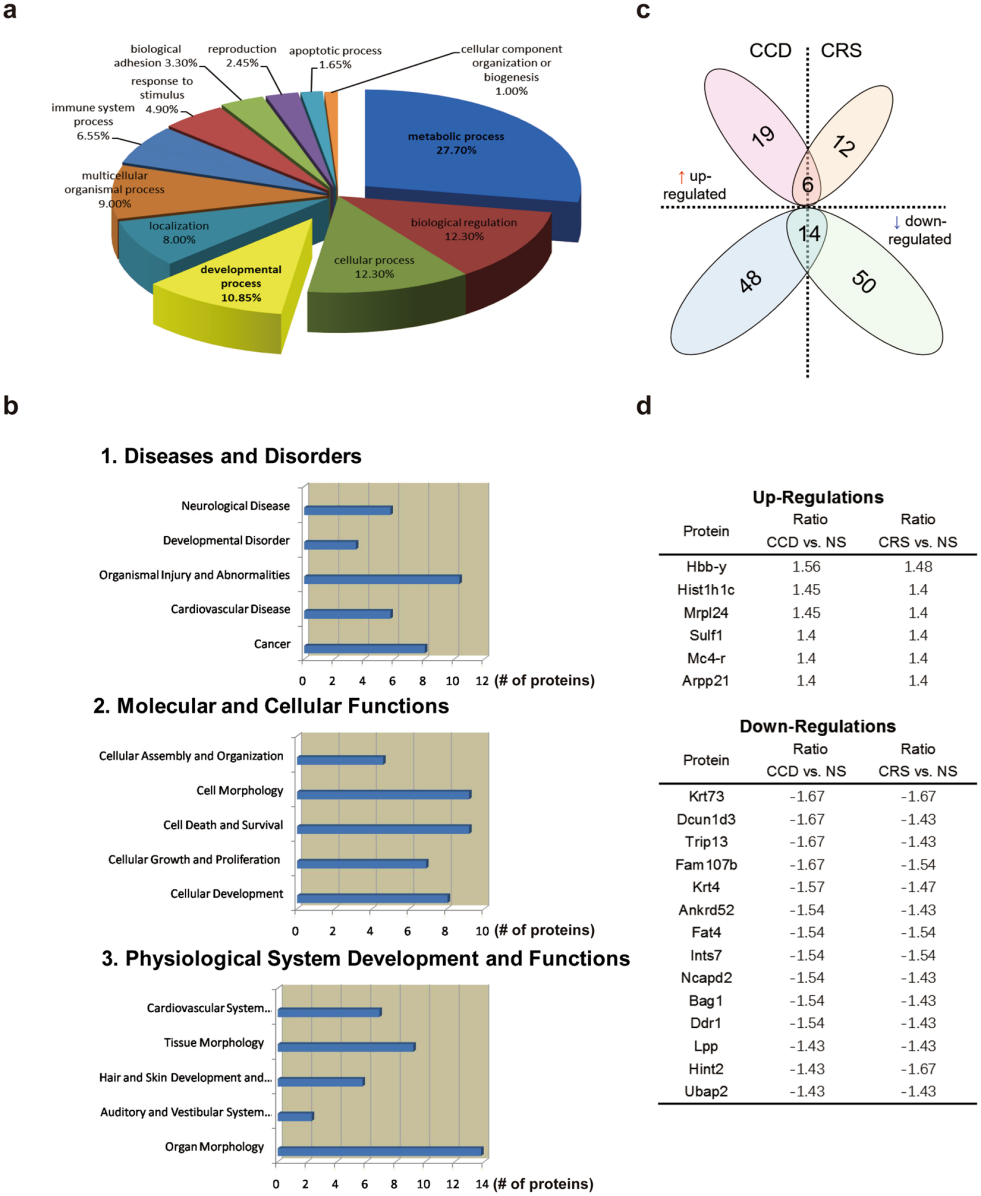
Analysis of the hypothalamic proteome of CCD F1 female mice using Gene Ontology (GO) and IPA databases. (**a**) Pie chart of biological processes analyzed from the GO analysis database. (**b**) IPA database showing the associations of the differentially expressed proteins in the hypothalamus of CCD F1 female mice with diseases/disorders (**b1**), molecular/cellular functions (**b**2) and physiological system development and functions (**b3**). (**c**) Cluster analysis of differentially expressed proteins in the CCD F1 and CRS F1 groups. Differential expression was established relative to NS F1 female mice. (**d**) The same trend of the protein list of CCD F1 and CRS F1 groups.

We surmise that chronic stress during the prenatal period may alter gene expression in the hypothalamus, an important locus for stress responses. To test this hypothesis, we compared the hypothalamic proteomes of 28-day-old CCD, CRS and NS F1 female mice using a quantitative mass spectrometric method called iTRAQ. From the 4394 total proteins that were detected, 87 showed differential expression between CCD and NS groups, and 82 between CRS and NS groups (Table S1), suggesting that the impact of these stress paradigms on hypothalamic protein expression is not widespread but more restricted to particular genes. By Gene Ontology (GO) analysis, metabolic processes represented the largest category of proteins that were differentially expressed between CCD and NS F1 mice (27.70%), followed by biological regulation (12.30%), cellular processes (12.30%), and developmental processes (10.85%) (Fig. 5a). A different distribution of GO categories was observed when comparing CRS and NS F1 mice, although metabolic processes remained the largest category (Fig. S4a). This suggests that the hypothalamic proteome of F1 mice is differentially affected by prenatal CCD and CRS stress. Proteins that were differentially expressed in the CCD and CRS groups were further analyzed by Ingenuity Pathway Analysis (IPA) and classified according to their associations with the categories diseases and disorders, molecular and cellular functions, and physiological system development and functions (Figs 5b and S4b). For each category, there was modest overlap between the top 5 subcategories (Figs 5b and S4b). We noted that a substantial proportion of proteins whose expression was altered in CCD F1 mice relative to NS controls was associated with the development and function of the central nervous system (e.g., *Myg1*, *Fat4*, *Syvn1*, *Rela*, *Sulf1*, *Pvalb*, *Sox1*, *Mapk3*, *Cbln3*) (Fig. 5b and Table S2). Changes in histone and histone modification-related proteins (e.g., *Hist1h1c*, *Ezh2*, *Akt1*, *Sap30*, *Rbbp4*, *Kdm3b*) mainly appeared in the CRS group (Table S2). Direct comparison between the 87 and 82 differentially expressed proteins in the CCD and CRS F1 groups, respectively, confirmed that there was little overlap (Fig. 5c and Table S2). For both prenatal stress paradigms, the majority of differentially expressed proteins in the hypothalamus were downregulated rather than upregulated (Fig. 5c). Of the 37 and 112 proteins that were increased or decreased in expression, respectively, by at least one of the stress paradigms, only 6 upregulated proteins and 14 downregulated proteins were common to both paradigms (Fig. 5d). The biological functions of these overlapping proteins were mainly divided into the GO categories of development (*Mrpl24*, *Fat4*, *Ints7*, *Trip13* and *Bag1*), metabolism (*Mc4-r* and *Arpp21*), cancer (*Fam107b*, *Ddr1* and *Lpp*), cell cycle (*Dcun1d3*), and associated with Alzheimer’s disease & Parkinson’s disease (*Ncapd2*) (Fig. 5d). Although prenatal CCD and CRS stress have similar effects on the mood-associated behaviors of F1 female mice, the proteomics data suggest that there may be differences between the two stress paradigms in terms of their underlying programming mechanisms.

### Prenatal CCD stress alters the expression of melanocortin 4 receptors in the hypothalamus of F1 mice in a sex-specific manner

**Figure 6:**
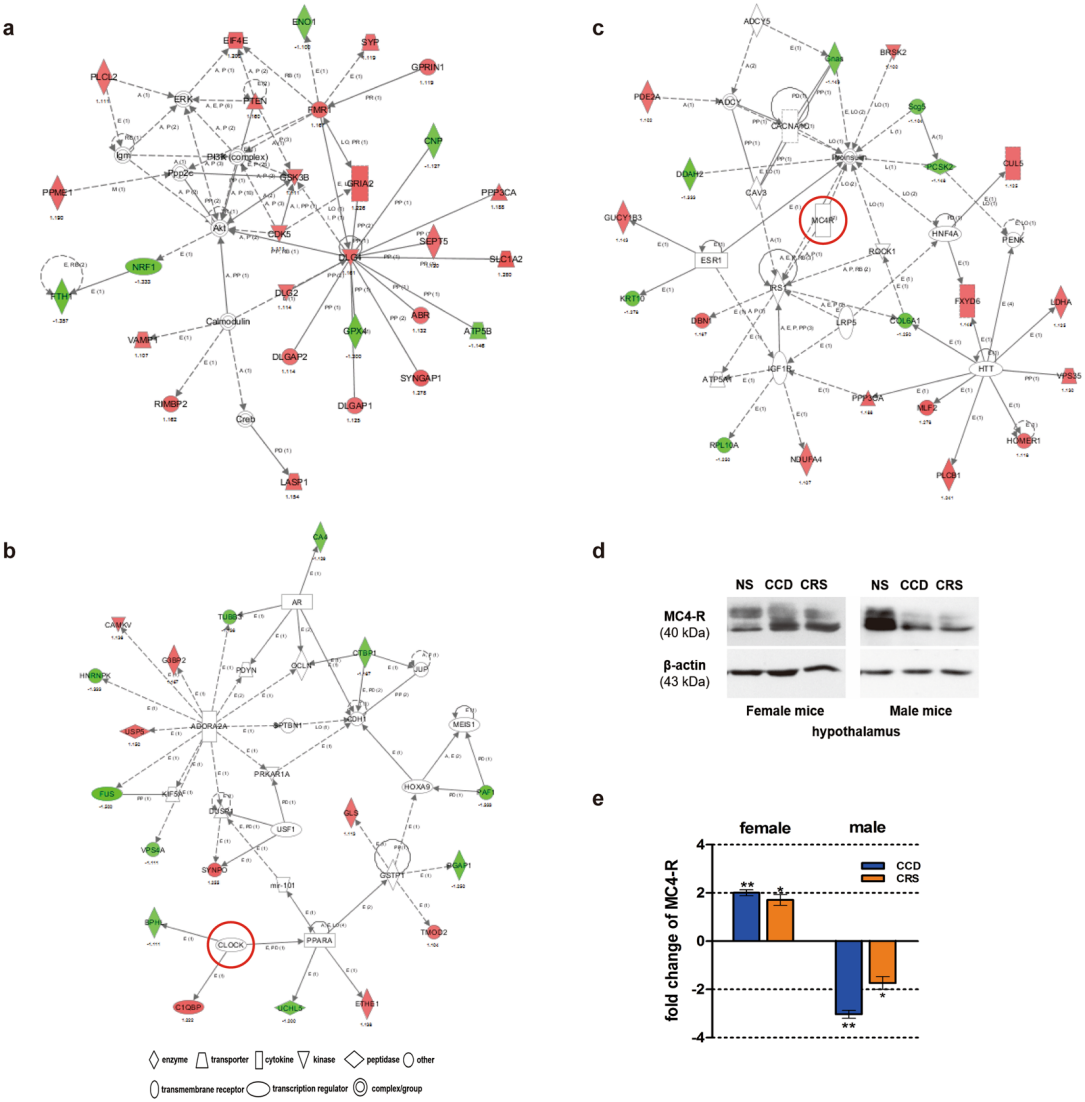
Protein interaction networks involving proteins that are differentially expressed in the hypothalamus of CCD F1 female mice. (**a**) Network of cell-to-cell signaling and interaction, nervous system development and function, and cell morphology. (**b**) Network of behavior, cardiovascular development and function, and organ development. (**c**) Network of endocrine disorders, metabolic disease, and endocrine system development and function. In panels a, b and c, red symbols represent up-regulated proteins, and green symbols represent down-regulated proteins. (**d**) Western blots showing the hypothalamic expression of type 4 melanocortin receptor (MC4-R) in female and male F1 mice from the CCD, CRS and NS groups. MC4-R is indicated by the red circle in network C. (**e**) Quantitative analysis of MC4-R protein expression based on Western blotting. *P < 0.05, **P < 0.01 vs. NS F1 group.

Next, we performed protein interaction network analysis on the proteins that were differentially expressed in the hypothalamus of CCD F1 female mice. The top three largest protein interaction networks were associated with: 1) cell-to-cell signaling and interactions, nervous system development and function, and cell morphology (Figs 2 and 6a) endocrine disorders, metabolic disease, and endocrine system development and function (Figs 3 and 6b) behavior, cardiovascular system development and function, and organ development (Fig. 6c). Interestingly, in the network associated with behavior (Fig. 6c), two interacting proteins of CLOCK were present, namely GLI-Kruppel family member GLI3 (BPH) and complement component 1 and q subcomponent binding protein (C1QBP).

To validate our mass spectrometric data, we assessed the expression of melanocortin 4 receptor (MC4-R) in the hypothalamus by Western blotting (Figs 6d,e and S2). MC4-R was one of the six proteins that were upregulated in expression by both prenatal CCD and CRS stress (Fig. 5d). In line with our mass spectrometric results, MC4-R protein levels were significantly upregulated in the hypothalamus of CCD and CRS F1 female mice relative to NS controls (Fig. 6d,e). However, it was markedly downregulated in CCD and CRS F1 male mice (Fig. 6d,e). We conclude that MC4-R expression is sensitive to prenatal stress, regardless of the nature of the stressor, and that the changes in its expression are sex-dependent.

## Discussion

The etiology of mood disorders such as major depression, anxiety and bipolar disorder remains unknown despite numerous hypotheses that attempt to explain their development. Inherited, environmental and lifestyle factors, including those that lead to circadian rhythm disturbances, have been associated with the pathogenesis of mood disorders^17,18^. Since the 1950s, disruptions or abnormalities in circadian rhythms and sleep/wake cycles have been documented in patients with mood disorders^19^, although it is unclear whether circadian rhythm disturbances cause mood disorders or vice versa. The increased rate of diagnosis of mood disorders may be partly attributed to our modern-day lifestyles, which include increasing exposure to artificial light at night, shift work, frequent travel across time zones, and an indoor lifestyle that prevents adequate exposure to the natural light of the sun. Studies using animal models have provided evidence that circadian rhythms and mood disorders are closely linked^20^. In our present study, we asked (1) whether circadian rhythm disturbance during the gestational period induces anhedonia- and depression-like behaviors in pregnant female mice, (2) whether *in utero* exposure to circadian rhythm disturbance leads to the development of these mood disorder-like behaviors in adolescent and adult mice that otherwise have not experienced postnatal stress, and (3) whether mice that experienced circadian disruption during fetal development also bear offspring that exhibit altered mood-associated behaviors.

To answer these questions, we conducted a transgenerational (F0, F1 and F2) assessment of mood disorder-like behaviors in response to exposure of pregnant F0 female mice to CCD stress. Our data suggest that CCD stress does not have deleterious effects on pregnant mice, as all successful mating resulted in full-term pregnancy and the birth of live pups. In contrast, mutation of the *Clock* gene has been shown to disrupt the estrus cycle and interfere with the maintenance of pregnancy^21^. Despite having no obvious effects on pregnancy maintenance, our CCD stress paradigm resulted in an extremely skewed sex ratio that was biased towards female. We observed mortality (2–3 pups/litter) in some, but not all, litters born to CCD F0 dams, which might contribute to the skewed sex ratio at weaning. The cause of neonatal mortality was not explored further, but might point to a potential deficit in lactation or nursing behavior. Assuming that plasma corticosterone remains elevated in the lactating F0 dams, it may affect maternal behavior. Interestingly, *Clock* mutant dams exhibit disrupted daily maternal behavior, resulting in reduced growth and survival of pups^22^. Furthermore, anxiety-related behaviors are increased in wild-type mice that have been reared by *Clock* mutant dams^23^.

In our study, CCD stress during the gestational period elicited anhedonia and depressive behavior in F0 females, as indicated by the reduction in sucrose preference and the increase in immobility time in the forced swim test. Neither the F1 nor the F2 generation experienced CCD stress postnatally, but they nevertheless displayed mood-associated behavioral phenotypes. Importantly, we noted phenotypic differences between the F1 and F2 generations and sexual dimorphism within the same generation. Sexual dimorphism was most apparent in the forced swim test in the F1 generation, where prenatal CCD stress resulted in depressive behavior in females but anti-depressive behavior in males. Previous studies have reported sex-dependent differences in behavior in various tests including the forced swim test, open field test, light/dark box test and elevated plus maze^24–26^. In those studies, sexual dimorphism was attributed to an interplay between an altered HPA axis and the hypothalamo-pituitary-gonadal axis, neurodevelopmental or neuro-plasticity changes in the fetal brain, or effects of maternal-derived immune factors on fetal brain circuits. In our study, we noted sex-specific differences in clock gene expression in the hypothalamus of CCD F1 mice that may also contribute to sexual dimorphism in forced swim test behavior. A recent study showed that immobility time in the forced swim test exhibits a diurnal rhythm that is different between male and female mice^27^. In the same study, the authors showed that these rhythms are altered by mutations in *Clock*, *Per1* or *Per2* in a sex-specific manner.

Another key issue is the phenotypic differences between the F1 and F2 generations. Prolonged immobility time in the forced swim test was observed for all groups in the F1 generation with the exception of CCD F1 males. However, in the F2 generation, regardless of sex or the stress paradigm used, all groups showed reduced immobility time. We speculate that prenatal stress leads to epigenetic reprogramming in the F1 generation that is transmitted to the F2 generation. However, we suggest that the F1 generation is additionally affected by alterations in maternal behavior of dams that had directly experienced gestational stress, whereas the F2 generation may be affected primarily by inherited epigenetic changes and not by differences in maternal care. There may be differences in maternal behavior of F0 and F1 dams, given that F0 dams (both CCD and CRS stressed) have elevated plasma corticosterone levels, whereas CCD and CRS F1 females do not. Another possible explanation is that the stress paradigms elicit both epigenetic reprogramming but also other non-heritable changes (e.g., neuroplasticity, hormone release) in the F1 generation that together determine the behavioral outcome. The F2 generation would inherit the epigenetic changes but not the non-heritable effects of prenatal stress, thereby leading to a difference in behavior. Yet another possibility comes from the observation that the HPA axis in the adolescence period is susceptible to programming by stressful experiences, leading to behavioural consequences later in life and potentially even influencing subsequent generations^28^. Hence, the stress of the forced swim test at day 28 in F1 females may additionally contribute to the inherited epigenetic changes in the F2 generation.

It should be noted that in our study design, we only tested for transmission of stress-induced mood disorder-like behaviors through the maternal line by mating CCD or CRS F1 female mice with NS F1 male mice. It remains to be determined whether males that experienced CCD or other stressors *in utero* bear offspring that show a similar susceptibility to developing mood disorder-like behaviors.

We hypothesize that gestational CCD stress affects the development of the fetal brain. We focused on the hypothalamus, because this brain region plays an important role in mood regulation and has been suggested to undergo fetal programming^29,30^. Only 4–5% of proteins detected in the hypothalamus of F1 females were altered by prenatal CCD or CRS stress, suggesting that the effects are restricted to specific genes. Furthermore, there was modest overlap between the two sets of differentially expressed hypothalamic proteins, potentially pointing to different programming mechanisms evoked by prenatal CCD and CRS stress.

For the CCD F1 mice, differentially expressed proteins included those that are related to nervous system development and metabolism (*Apoa1*, *Mc4-r*). In addition, some differentially expressed proteins are associated with signaling pathways that have been implicated in mood disorders^31^, including the glucocorticoid receptor (GR) signaling pathway (RELA, BAG1, NCOR and RNA Pol II). As one of the most important effectors of the HPA axis, glucocorticoids are essential for the development of neural tissues^32^. The ability of the prenatal glucocorticoid milieu to program the HPA axis has been demonstrated in several species^33^. In addition to changes in hypothalamic protein expression, plasma corticosterone levels were elevated in 28-day-old CCD F1 mice compared with age-matched NS F1 controls. Prenatal stress and overexposure to glucocorticoids during development may be associated with an increased susceptibility to a number of diseases in adulthood including depression, anxiety and other neuropsychiatric disorders^34^. In addition to potentially regulating glucocorticoid production^35^, MC4-R plays an important regulatory role in energy balance and body weight^36,37^. Abnormal body weight is often a common phenotype in animal models of mood disorders. It should be noted that MC4-R upregulation was common to both prenatal CCD and CRS stress, suggesting a point of convergence for different prenatal stressors. Our results strongly suggest that gestational CCD stress can alter the HPA axis of the F1 generation even in the absence of postnatal stress. In light of studies showing that GR function is regulated by various core clock proteins^38^, circadian rhythm disturbance-induced dysfunction of the HPA axis may be a key mechanism underlying mood disorder-like behaviors in the F1 generation. Whether the changes in plasma corticosterone levels, clock gene expression in the SCN, and hypothalamic protein expression persist to the F2 generation remains to be determined.

In the case of prenatal CRS stress, we observed altered expression of proteins related to histone and histone modification. Epigenetic modifications of DNA and chromatin structure induced by environmental factors, including stress, may contribute to the complex phenotypes of neuropsychiatric disorders^39,40^. A recent study found that gestational CRS promotes gene-specific DNA methylation to influence adult depression-like behavior^41^. Along these lines, we noted differential expression of Ezh2 and Akt1, which are implicated in histone methylation.

Our animal study aligns well with clinical evidence showing the impact of maternal experiences on the developing fetus. Human mothers who experience stress during pregnancy have children who are more likely to exhibit emotional or cognitive problems, including an increased risk of attentional deficit/hyperactivity disorders, anxiety, and delayed language acquisition^5^. The fetal brain appears to be highly sensitive to perinatal programming, which may involve a permanent reorganization of neural circuits^42–44^. Epigenetic mechanisms are suggested to underlie perinatal programming^1,45,46^. Likewise, epigenetic changes may explain why gestational stress can lead to mood disorder-like behaviors over multiple generations.

In conclusion, we found that prenatal CCD stress can lead to mood disorder-like behaviors in the F1 and F2 generations. These behaviors in F1 mice are associated with perturbations in core circadian clock gene expression in the SCN, and altered expression of hypothalamic proteins and mood-relevant signaling pathways. Changes in plasma corticosterone levels may also contribute to the pathogenesis of mood disorders in the F1 offspring. Our study provides evidence that prenatal CCD stress-induced mood-associated behavioral perturbations persist to the second filial generation, thereby highlighting the potential detrimental effects that chronic shift work by pregnant women may have on their children and grandchildren.

## Materials and Methods

### Animals

C57BL/6 J mice, age 6–7 weeks, were purchased from the Experimental Animal Center, Southwest Medical University (Luzhou). Mice were housed in a room with an ambient temperature of 23 ± 2 °C and a 12:12 h light-dark (LD) cycle where lights-on was set at 8:00 am local time and lights-off at 20:00 pm (light intensity 200 lux). Mice were given *ad libitum* access to regular rodent chow and water. Nulliparous female mice (8 weeks old) were housed with age-matched male mice in a 2:1 ratio. The day of discovery of a vaginal plug was designated as gestational day (G) 0. Pregnant mice were randomly divided into three groups according to treatment: (1) CCD group, (2) CRS group, and (3) NS control group. Pregnant mice were housed individually from G0 until delivery (19 to 21 days later). Body weights of pregnant mice were measured at G0 and G18. All successful mating resulted in the birth of live pups, indicating that there was no spontaneous abortion of entire litters under any of the stress paradigms. We also did not observe any stillbirths. Pups were weaned on postnatal day (PND) 21, and the number of female and male pups for each litter were noted at the time of weaning. We noticed that of the 10 CCD F0 dams used in the study, some cannibalized 2 or 3 pups in their litters within a few days of birth, potentially accounting for the skewed sex ratio. The animal use procedures were approved by the Life Ethics Committee of Southwest Medical University and were conducted in compliance with the U.S. National Institutes of Health Guidelines for the Care and Use of Laboratory Animals (NIH Publication 85–23, revised 1985).

### Experimental design

A total of 309 mice over three generations were bred and used in this study. F0 female mice were subjected to CRS or CCD stress from G0 to G18, or were maintained under non-stressed (NS) conditions throughout the gestational period (n = 10 in each group). The CCD paradigm involved delaying the 12:12 LD cycle by 8 hours every 2 days from G0 to G18.

F1 progeny were weaned at PND 21 and split into female (n = 33) and male (n = 18) subgroups. At PND 84, CCD and CRS F1 female mice were mated with NS F1 male mice. F2 progeny were likewise weaned at PND 21, split into female (n = 15) and male (n = 15) subgroups, and treated in the same manner as the F1 generation. Behavioral tests were performed on F1 mice at PND 28, 56 and 84, and on F2 mice at PND 28 and 56.

### Procedure for chronic restraint stress

F0 female mice were subjected to CRS for 19 consecutive days from G0 to G18. Mice were individually placed into a plastic tube (length 10 cm, inner diameter 3 cm) with holes in the bottom and walls of the tube to allow ventilation. Three sessions of body restraint, each lasting 30 min, were administered at 8:00 am, 12:00 pm and 16:00 pm.

### Behavioral tests

#### Forced swim test

The forced swim test was used to evaluate depression-related responses according to the method reported by Porsolt *et al*.^47^. The test period was ZT 1 to ZT 3. Mice were placed individually into glass cylinders (height 25 cm, diameter 10 cm) containing 10-cm deep water maintained at 23–25 °C and observed for 6 min. The total duration of immobility time in the final 4 min of the test was measured. The mouse was judged to be immobile when it remained floating passively in the water.

#### Sucrose preference test

For the F0 generation, pregnant mice were presented with two identical bottles containing 2% sucrose or water in their home cage, and consumption was measured over a 24-hour period starting at G0 and G18. For the F1 and F2 generations, mice were habituated to the presence of two drinking bottles containing 2% sucrose or water in their home cage for 48 hours before the test day. On test day, mice were deprived of fluids and food for 4 hours from 13:00 pm to 17:00 pm (ZT 5 to ZT 9) before being presented with two identical bottles containing 2% sucrose or water for 1 hour. Bottles were weighed before and after the 1-hour interval (or after the 24-hour interval for F0), and sucrose preference (%) was calculated with the following equation: sucrose preference % = weight of ingested sucrose solution/[weight of ingested sucrose solution + weight of ingested water] × 100%. Reduction in sucrose preference (%) is an indicator of anhedonia, or a lack of interest in rewarding stimuli.

CRS and CCD stress were administered to F0 mice only, but mice from all generations (F0, F1 and F2) were assessed in the sucrose preference test and forced swim test. Mice were denoted by their generation, sex and the stress paradigm (CCD, CRS, or NS) that they experienced while pregnant (in the case of F0) or that the F0 females from which they were derived experienced (in the case of F1 and F2).

### Tissue sampling

Blood samples were collected from the tail vein of F1 female mice on PND 28 and 56 between 2:00 pm and 6:00 pm under anesthesia with 1.5% sodium pentobarbital (0.07 ml/10 g body weight). To separate serum from whole blood, samples were centrifuged at 3,000 rpm for 15 min and the serum was stored at −80 °C until use.

For RNA and protein experiments, brains were harvested from F1 female mice at PND 28 and 56, and from F1 male mice at PND 28. For clock gene expression studies, F1 female mice were killed by decapitation at Zeitgeber time (ZT) 0, 4, 8, 12, 16 and 20 (n = 3 per time point per group) under white light during the light phase of the LD cycle or under dim red light during the dark phase. For tissue harvests in the dark phase, eyes were covered with black electrical tape prior to brain dissection. Individual SCN tissue was collected using a trocar with an inner diameter of 1.04 mm. The whole brain was placed ventral side facing up, and the trocar was inserted from the optic chiasm through the cerebral cortex. After removing the tissue core from the trocar, SCN tissue was isolated by cutting 0.5 mm above the optic chiasm. Hypothalamic tissues were dissected from the brain using a scalpel and tweezer. All tissues were snap frozen in liquid nitrogen.

For mass spectrometry experiments, brains were harvested from F1 female mice belonging to the CCD, CRS and NS groups at PND 28 (n = 3 in each group). Hypothalamic tissues were dissected and snap frozen in liquid nitrogen.

### Sample preparation for mass spectrometric analyses

Individual hypothalamic tissue samples were homogenized in 2 ml lysis buffer (8 mol/L urea, 4% CHAPS and 100 mmol/L NH_4_HCO_3_ mixed with fresh proteinase inhibitor) using a syringe fitted with a 32 G gauge needle. Homogenates were sonicated on ice with 5 pulses of 15–20 sec each, with intervals of 1 min between pulses. Protein lysates were centrifuged at 14,000 rpm for 20 min at 4 °C, and the supernatant was collected and stored at −80 °C. Protein concentration was determined with the Bradford method. Protein concentration and integrity were confirmed by SDS polyacrylamide gel electrophoresis and Coomassie brilliant blue staining. Solid-phase extraction (SPE) method was used to separate, purify and concentrate protein samples. Proteins were digested overnight at 37 °C in sequencing-grade modified trypsin (Promega, Madison, WI, USA). Peptides were extracted using 50% acetonitrile in 5% acetic acid solution under ultrasound sonication, then dried and concentrated using a vacuum concentrator (Eppendorf AG, Hamburg, Germany).

### Isobaric tagging for relative and absolute quantitation (iTRAQ) and LC-MS/MS

For iTRAQ experiments, peptide samples were prepared from hypothalamic tissues of CCD F1, CRS F1 and NS F1 female mice (n = 3 per group), and labelled with iTRAQ reagents according to the manufacturer’s protocol (AB Sciex). Peptides from NS, CCD and CRS samples were labelled with iTRAQ reagents 114, 115 and 116, respectively. The three labeled samples were combined and lyophilised, and subsequently separated by strong cation exchange (SCX) chromatography on a Dionex Ultimate nano-LC system, using a polysulfoethyl A column (2.1 mm i.d. × 150 mm, 5 µm, 300 Å, Phenomenex). The sample was loaded and washed using 25% (v/v) acetonitrile and 10 mmol/L phosphoric acid for 20 min at 200 µl/min. Peptides were eluted with a linear gradient of 0–500 mmol/L KCl in 25% (v/v) acetonitrile and 10 mmol/L phosphoric acid at 200 µl/min and fractions collected at 1 min intervals. Fractions were lyophilised *in vacuo*, resuspended in 50 µl of MilliQ water and separated by nano-RP-LC using a PepMap C_18_ guard column (5 mm × 300 µm i.d., Dionex) and a C_18_ reverse phase PepMap column (150 mm × 75 µm i.d., Dionex). The resulting peptide samples were electrosprayed into a Q-tof mass spectrometer (Waters Ltd).

### Data processing

Scaffold Q + software (Scaffold version 4.2.1., Proteome Software Inc., Portland, OR) was used to quantify Label Based Quantitation (iTRAQ) peptides and to identify proteins. Peptide identifications were accepted if they could be established at greater than 99.0% probability to achieve an FDR less than 1.0% by the Scaffold Local FDR algorithm. Protein identifications were accepted if they could be established at greater than 85.0% probability to achieve an FDR less than 1.0% and contained at least 1 identified peptide. Protein probabilities were assigned by the Protein Prophet algorithm^48^. Proteins that contained similar peptides and could not be differentiated based on MS/MS analysis alone were grouped to satisfy the principles of parsimony. Proteins sharing significant peptide evidence were grouped into clusters. Channels were corrected by the matrix (0.000, 0.01000, 0.925, 0.0630, 0.00200), (0.000, 0.0200, 0.919, 0.0600, 0.001000), (0.000, 0.0300, 0.920, 0.0490, 0.001000) and (0.001000, 0.0400, 0.920, 0.0380, 0.001000) in all samples according to the algorithm described in i-Tracker^49^. Acquired intensities in the experiment were globally normalized across all acquisition runs. Individual quantitative samples were normalized within each acquisition run. Intensities for each peptide identification were normalized within the assigned protein. The reference channels were normalized to produce a 1:1 fold change. All normalization calculations were performed using medians to multiplicatively normalize data.

### Database search and analysis

Raw files were processed and analyzed by MaxQuant (version 1.3.0.5) against the mouse international protein index protein sequence database (IPA Mouse, version 3.75), including commonly observed contaminants. Cysteine carbamidomethylation was selected as a fixed modification, and the methionine oxidation and protein N-terminal acetylation were set for variable modifications. Enzyme specificity was set to trypsin.

The protein group file was imported into Perseus (version 1.3.0.4) for statistical analysis of the data. The raw dataset (4394 proteins) was filtered to include only proteins with a minimum peptide ratio count of 1.4, resulting in stringently quantified datasets of 4001 and 3930 proteins in the CCD and CRS groups, respectively. One-way ANOVA was used to analyze this stringent dataset for temporal regulation, with *p* values < 0.05 indicating statistical significance. For the hierarchical clustering analysis, median value of logarithmized values for the normalized L/H ratio of each protein profile was performed after z-score normalization of the data within Euclidean distances. Based on the ratio in group, expression levels that are higher or lower than the ratio count of 1.4 (including 1.4) were considered to be significantly different between two groups (CCD F1 *vs*. NS F1 (87 proteins) and CRS F1 *vs*. NS F1 (82 proteins)) (Supplementary Table 1). The datasets of 87 and 82 significantly altered proteins were mapped and summarized with IPA and GO analyses. Canonical pathways analyses were performed with *p* value of 0.05 and networks were displayed with minimum significant score of 11. Biological process analysis was achieved using IPA.

### Reverse transcription-quantitative PCR (RT-qPCR)

Total RNA was extracted from individual SCN tissues using Trizol reagent (Invitrogen) according to manufacturer’s instruction. RNA concentration and purity were determined using the NanoDrop 2000 (Thermo Fisher, USA), and RNA integrity was confirmed by agarose gel electrophoresis. cDNA synthesis was performed using the TransScript First-Strand cDNA Synthesis Super Mix kit (TransGen Biotech Co., China) and 20 ng of total RNA. qPCR reactions were prepared with SYBR Premix Ex Taq (TaKaRa) and run on the CFX96 Real-Time qPCR System (Bio-Rad, USA). Primer sequences were as follows: *Clock* forward, 5′-TTAGATCACAGGGCACCACC-3′; *Clock* reverse, 5′-GTGCTCGTGACATTTTGCCA-3′; *Bmal1* forward, 5′-GGCCTTCATTGCACCTTCCTT-3′; *Bmal1* reverse, 5′-GAACCGGAGAGTAGGTCGGT-3′; *Per1* forward, 5′-TCGAAACCAGGACACCTTCTCT-3′; *Per1* reverse, 5′-GGGCACCCCGAAACACA-3′; *Per2* forward, 5′-CATTGAACTTGAGACTGAGGT-3′; *Per2* forward, 5′-AAGGGAACACACTGAGAGGAT-3′; *GAPDH* forward, 5′-CAAGGTCATCCATGACAACTTTG-3′; *GAPDH* reverse, 5′-GTCCACCACCCTGTTGCTGTAG-3′.

### Western blotting

SCN and hypothalamic tissues were homogenized on ice in RIPA buffer containing protease inhibitors. Homogenized tissues were incubated on ice for 30 min and centrifuged at 12,000 rpm for 15 min. Supernatant was collected and stored at −80 °C. Protein concentration was measured using the Bradford assay. Protein lysates were mixed with SDS loading buffer to 1 × concentration, heated to 95 °C for 5 min, and centrifuged for 1 min at 12,000 rpm. Lysates (20 μg/well) were electrophoresed in a 10% SDS polyacrylamide gel for approximately 2 h at 100 V at room temperature (RT) and electroblotted onto polyvinylidene fluoride membrane for 30 minutes at 25 V RT. Membranes were blocked in 5% skim milk in Tris buffered saline with 0.1% Triton X-100 (TBS-T) for 1 h at RT, followed by incubation with rabbit anti-MC4-R (1:500, SC-28992, Santa Cruz Biotechnology) in blocking solution at 4 °C overnight. The next day, membranes were washed in TBS-T and incubated for 2 h at RT with secondary antibody in blocking solution. Blots were developed using an enhanced chemiluminescence detection kit (Amersham ECL, USA), and images were captured using ImageQuant LAS 4000 mini (GE Healthcare, USA). Membranes were stripped, redeveloped with ECL to verify stripping efficiency, and reprobed with antibodies against β-actin (1:5000, 061M4808, Sigma-Aldrich). Blots were quantified using the “Analyst” function in Quantity One 4.4. Values are presented as fold-change of the protein normalized to relative abundance of β-actin from 3 mice per time point.

### Enzyme linked immunosorbent assay (ELISA)

Serum corticosterone levels were measured with a commercial ELISA kit (CEA540Ge 96 Test, USCN, USA) according to manufacturer’s instructions.

### Statistical analysis

All values were expressed as mean ± standard deviation (SD). Student’s tests or two-way ANOVA were performed for comparison of group differences where appropriate. Prior to the use of parametric statistics, we ensured that data were normally distributed (Shapiro-Wilk test). Differences were considered significant at *P* < 0.05.

## Electronic supplementary material

Supplementary information

## References

[1] Babenko O, Kovalchuk I, Metz GA (2015). Stress-induced perinatal and transgenerational epigenetic programming of brain development and mental health. Neurosci Biobehav Rev..

[2] Franklin TB (2010). Epigenetic transmission of the impact of early stress across generations. Biol. Psychiatry..

[3] Van den Bergh BR, Mulder EJ, Mennes M, Glover V (2005). Antenatal maternal anxiety and stress and the neurobehavioural development of the fetus and child: links and possible mechanisms. A review. Neurosci Biobehav Rev..

[4] Talge NM, Neal C, Glover V (2007). Antenatal maternal stress and long-term effects on child neurodevelopment: how and why?. J. Child Psychol Psychiatry..

[5] Cardwell MS (2013). Stress: pregnancy considerations. Obstet Gynecol Surv..

[6] Khashan AS (2012). Prenatal stress and risk of asthma hospitalization in the offspring: a Swedish population-based study. Psychosom Med..

[7] Zhang X, Sliwowska JH, Weinberg J (2005). Prenatal alcohol exposure and fetal programming: effects on neuroendocrine and immune function. Exp. Biol. Med..

[8] Barker DJ (1999). Fetal origins of cardiovascular disease. Ann Med..

[9] Liu C, Chung M (2015). Genetics and epigenetics of circadian rhythms and their potential roles in neuropsychiatric disorders. Neurosci. Bull..

[10] Kronfeld-Schor N, Einat H (2012). Circadian rhythms and depression: Human psychopathology and animal model. Neuropharmacology..

[11] Baba M (2015). Analysis of salivary cortisol levels to determine the association between depression level and differences in circadian rhythms of shift-working nurses. J. Occup. Health..

[12] Flo E (2012). Shift work disorder in nurses—assessment, prevalence and related health problems. PloS One..

[13] Eldvik, M. F., Flo, E., Moen, B. E., Pallesen, S. & Bjorvatn, B. Insomnia, excessive sleepiness, excessive fatigue, anxiety, depression and shift work disorder in nurses having less than 11 hours in–between shifts. *PloS On*e. 8, e70882; 10.1371/journal.pone.0070882. eCollection 2013 (2013). 10.1371/journal.pone.0070882 PMC3744484 23976964

[14] Francesco M, Patricia T, Stefania M, Ferdinando N, Alessandro G (2012). Pharmacological activation of group-II metabotropic glutamate receptors corrects a schizophrenia-like phenotype induced by prenatal stress in mice. Neuropsychopharm acology..

[15] Matrisciano F (2013). Epigenetic modifications of GABAergic interneurons are associated with the schizophrenia-like phenotype induced by prenatal stress in mice. Neuropharmacology.

[16] Weaver DR (1998). The suprachiasmatic nucleus: A 25-year retrospective. J. Biol. Rhythems..

[17] Bunney WE, Bunney BG (2000). Molecular clock genes in man and power animals: possible implications for circadian abnormalities in depression. Neuropsychopharmacology..

[18] Rumble ME, White KH, Benca RM (2015). Sleep Disturbances in mood disorders. Psychiatr. Clin. North Am..

[19] Wirz-Justie A (2006). Biological rhythm disturbances in mood disorders. Int. Clin. Psychopharmacol..

[20] McClung CA (2013). How Might Circadian Rhythms Control Mood? Let Me Count the Way. Biol. Psychiatry..

[21] Miller BH (2004). Circadian clock mutation disrupts estrous cyclicity and maintenance of pregnancy. Curr Biol..

[22] Hoshino K, Wakatsuki Y, ligo M, Shibata S (2006). Circadian Clock mutation in dams disrupts nursing behavior and growth of pups. Endocrinology..

[23] Koizumi, H., Kurabayashi, N., Watanabe, Y., & Sanada, K. Increased anxiety in offspring reared by circadian Clock mutant mice. *PLoS On*e. 8, e66021. 10.1371/journal.pone.0066021. Print 2013 (2013). 10.1371/journal.pone.0066021 PMC3680406 23776596

[24] Grundwald NJ, Brunton PJ (2015). Prenatal stress programs neuroendocrine stress responses and affective behaviors in second generation rats in a sex-dependent manner. Psychoneuroendocrinology..

[25] Gilman SE (2016). Prenatal immune programming of the sex-dependent risk for major depression. Transl Psychiatry.

[26] Rayen I, Gemmel M, Pauley G, Steinbusch HW, Pawluski JL (2014). Developmental exposure to SSRIs, in addition to maternal stress, has long-term sex-dependent effects on hippocampal plasticity. Psychopharmacology (Berl)..

[27] Li N, Xu Y, Chen X, Duan Q, Zhao M (2015). Sex-specific diurnal immobility induced by forced swim test in wild type and clock gene deficient mice. Int J Mol Sci..

[28] Tzanoulinou S, Sandi C (2017). The Programming of the Social Brain by Stress During Childhood and Adolescence: From Rodents to Humans. Curr Top Behav Neurosci..

[29] Barrett ES, Swan SH (2015). Stress and androgen activity during fetal development. Endocrinology..

[30] Goldstein JM, Handa RJ, Tobet SA (2014). Disruption of fetal hormonal programming (prenatal stress) implicates shared risk for sex differences in depression and cardiovascular disease. Front Neuroendocrinol..

[31] Biegon A, Reches A, Snyder L, McEwen BS (1983). Serotonergic and noradrenergic receptors in the rat brain: modulation by chronic exposure to ovarian hormones. Life Sci..

[32] Meter JS (1985). Biochemical effects of corticosteroids on neural tissues. Physiol Rev..

[33] Matthews SG (2002). Early programming of the hypothalamic-pituitary-adrenal axis. Trends Endocrinol Metab..

[34] Hiroi R, Carbone DL, Zuloaga DG, Bimonte-Nelson HA, Handa RJ (2016). Sex-dependent programming effects of prenatal glucocorticoid treatment on the developing serotonin system and stress-related behaviors in adulthood. Neuroscience..

[35] Doghman M (2004). Agouti-related protein antagonizes glucocorticoid production induced through melanocortin 4 receptor activation in bovine adrenal cells: a possible autocrine control. Endocrinology..

[36] Grill HJ, Ginsberg AB, Seeley RJ, Kaplan JM (1998). Brainstem application of melanocortin receptor ligands produces long-lasting effects on feeding and body weight. J Neurosci..

[37] Fisher, S. L., Yagaloff, K. A. & Burn, P. Melanocortin-4receptor: a novel signalling pathway involved in body weight regulation. *Int Obes Relat Metab Disord*. Suppl 1, 54–48 (1999). 10.1038/sj.ijo.0800796 10193863

[38] Albrecht, U. Molecular mechanisms in mood regulation involving the circadian clock. Front Neurol. **8**, 30. 10.3389/fneur.2017.00030. eCollection 2017 (2017). 10.3389/fneur.2017.00030 PMC5293817 28223962

[39] Dong E (2015). Brain-derived neurotrophic factor epigenetic modifications associated with schizophrenia-like phenotype induced by prenatal stress in mice. Biol Psychiatry..

[40] Zhang TY, Labonté B, Wen XL, Turecki G, Meaney MJ (2013). Epigenetic mechanisms for the early environmental regulation of hippocampal glucocorticoid receptor gene expression in rodents and humans. Neuropsychopharm acology..

[41] Jiao J, Opal MD, Dulawa SC (2013). Gestational environment programs adult depression-like behavior through methylation of the calcitonin gen-related peptide gene. Mol Psychiatry..

[42] Ralevski A, Horvath TL (2015). Developmental programming of hypothalamic neuroendocrine systems. Front Neuroendocrinol..

[43] Davis EP, Pfaff D (2014). Sexually dimorphic responses to early adversity: implications for affective problems and autism spectrum disorder. Psychoneuroendocrinology..

[44] Kim, D. J. *et al*. Prenatal maternal cortisol has sex-specific associations with child brain network properties. *Cereb. Cortex*. 10.1093/cercor/bhw303 (2016). 10.1093/cercor/bhw303 PMC6084613 27664961

[45] Keverne EB (2014). Significance of epigenetics for understanding brain development, brain evolution and behavior. Neuroscience..

[46] Lucassen PJ (2013). Perinatal programming of adult hippocampal structure and function; emerging roles of stress, nutrition and epigenetics. Trends Neurosci..

[47] Porsolt RD, Bertin A, Jalfre M (1977). Behavioral despair in mice: a primary screening test for antidepressants. Arch. Int. Pharmacodyn. Ther..

[48] Nesvizhskii AI, Keller A, Kolker E, Aebersold R (2003). A statistical model for identifying proteins by tandem mass spectrometry. Anal. Chem..

[49] Shadforth IP, Dunkley TP, Lilley KS, Bessant C (2005). i-Tracker: for quantitative proteomics using iTRAQ. BMC Genomics..

