# Supplementary material for: Environmental perturbation of the circadian clock during pregnancy leads to transgenerational mood disorder-like behaviors in mice
Source: Sci Rep. 2017 Oct 3;7:12641. doi: 10.1038/s41598-017-13067-y (PMC5626699; doi:10.1038/s41598-017-13067-y)
Supplement: Supplementary file 1 — Supplementary information [file 41598_2017_13067_MOESM1_ESM.pdf]

Supplementary information

Environmental perturbation of the circadian clock during pregnancy leads to transgenerational mood disorder-like behaviors in mice

Peng Zhang[*1], Guang Li[1], Hui Li[1], XiaoQiu Tan[*1], Hai-Ying Mary Cheng[*2]

1. Key Laboratory of Medical Electrophysiology, Ministry of Education, Institute of Cardiovascular Medicine; Collaborative Innovation Center for Prevention and Treatment of Cardiovascular Disease of Sichuan Province; Southwest Medical University, Luzhou 646009, China. 2. Department of Biology, University of Toronto Mississauga, 3359 Mississauga Road, Mississauga, ON L5L 1C6, Canada

* Corresponding author (email: zhangpeng1440@163.com)

Supplementary Figures and Legends

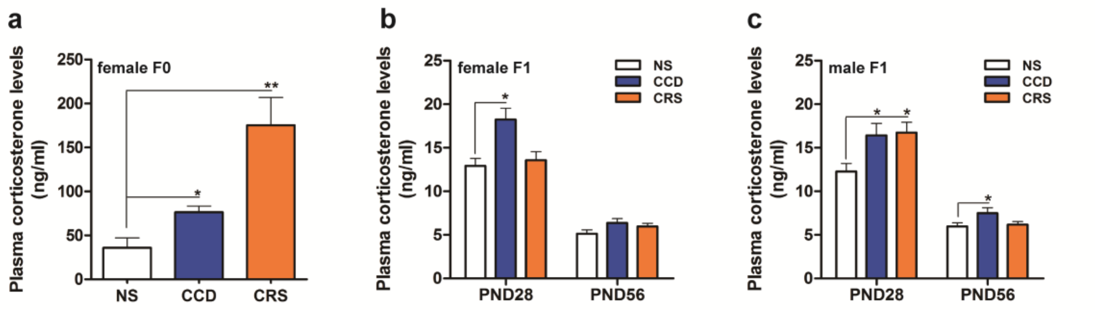

Supplementary Figure S1. Plasma corticosterone levels of F0 and F1 mice that had been exposed to different stresses *in utero*. (**a**) Plasma corticosterone levels of pregnant F0 mice at G18. (**b, c**) Plasma corticosterone levels of F1 female (b) and male (b) mice at PND 28 and PND 56. * $P < 0.05$ vs. NS F1 mice, n = 10 per group.

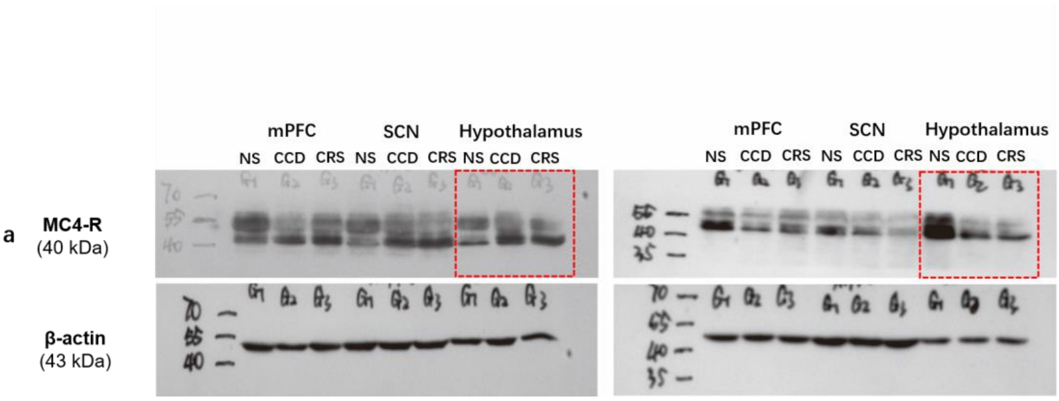

Supplementary Figure S2. The full-length blots for MC4-R and β-actin in medial prefrontal cortex (mPFC), SCN and hypothalamus of CCD, CRS and NS F1 mice at ZT 8. The images of MC4-R (**a**) and β-actin (**b**) after ECL detection were captured using ImageQuant LAS 4000 mini and a series of exposures were taken. Some bands appear non-specific. Only the bands with the relevant molecule size were cropped and presented in Fig. 6.

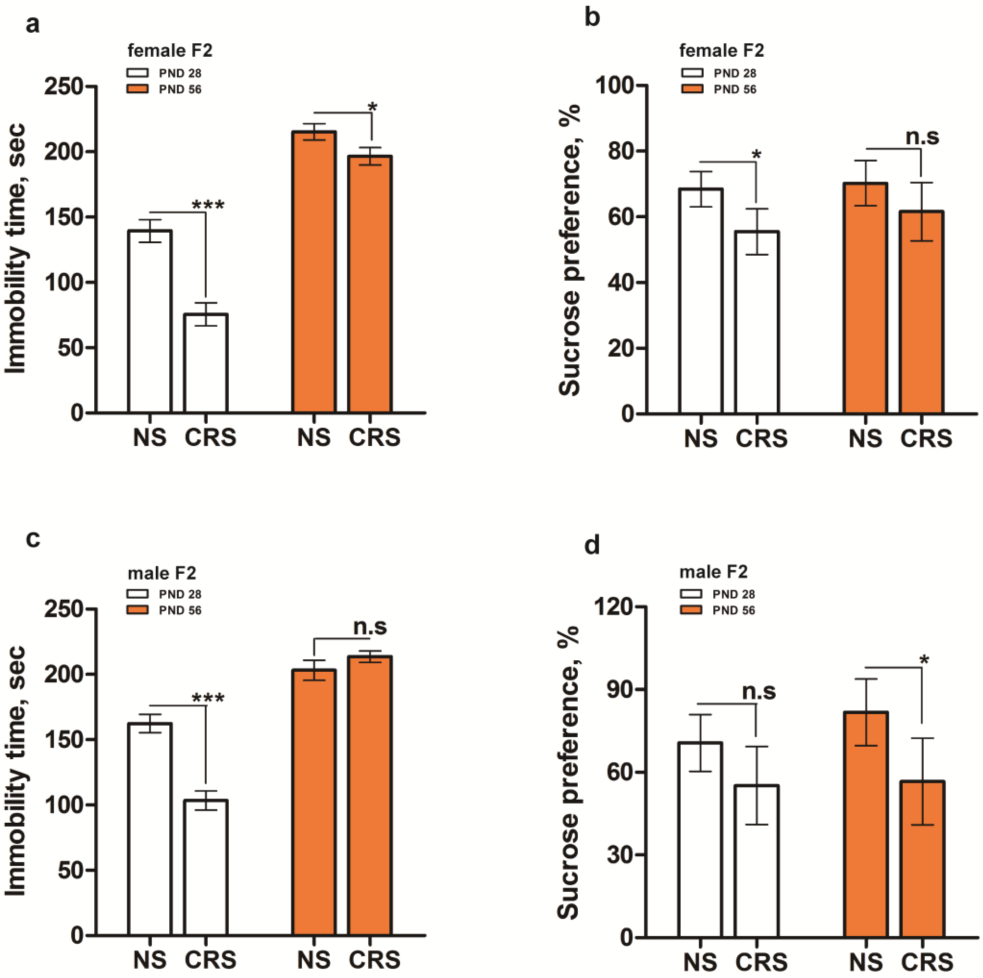

Supplementary Figure S3. Gestational CRS stress in the F0 generation leads to mood disorder-like phenotypes in the F2 generation. (**a, c**) FST immobility times of female (a) and male (c) F2 mice at PND28 and 56. (**b, d**) Sucrose preference of female (b) and male (d) F2 mice at PND28 and 56. **P* < 0.05, *** *P* < 0.001 *vs.* sex-matched NS F2 group, n = 15 per group.

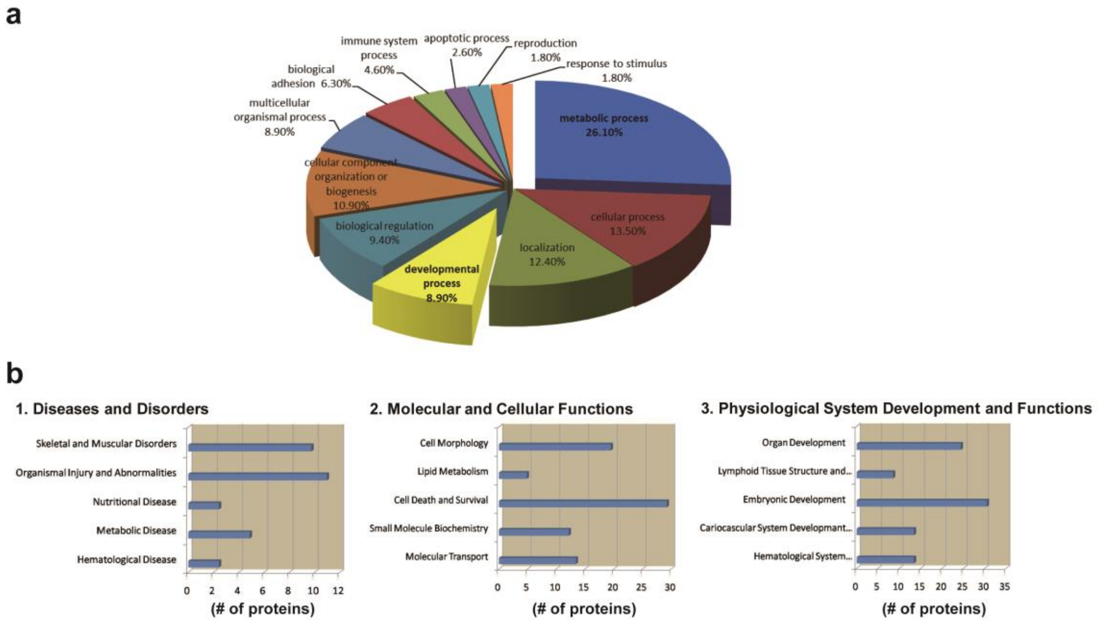

Supplementary Figure S4. Analysis of the hypothalamic proteome of CRS F1 female mice using Gene Ontology (GO) and IPA databases. (**a**) Pie chart of biological processes analyzed from the GO analysis database. (**b**) IPA database showing the associations of the differentially expressed proteins in the hypothalamus of CRS F1 female mice with diseases/disorders (b1), molecular/cellular functions (b2) and physiological system development and functions (b3).

Supplementary Table 1 & 2

Supplemental Table1. Standard of data screening.

	Fold changes	F1-CCD vs. F1-NC Protein number	F1-CRS vs. F1-NC Protein number
**Total protein**		4394	4394
**CV*[1] < 3%**		4163	4120
**Repeatability ratio*[2]**	0.714286<, <1.4	4001	3930
**Groups ratio*[3]**	>1.4, <0.714286	87	82
**False positive rate**		3.9%	4.6%

*1. CV is the standard deviation / average value of absolute quantitative value of two tests.

*2. Analysis of the two mass spectrometry repeated the same sample: two results of Ratio≤ 5%.

*3. Comparison of different sample absolute quantitative differences: the false positive rate of less than 5%.

Supplemental Table2. Up-and Down-regulations protein of CCD and CRS F1 female mice.

Up-Regulations				Down-Regulations				Down-Regulations			
CCD vs. NS		CRS vs. NS		CCD vs. NS		CRS vs. NS		CCD vs. NS		CRS vs. NS	
Protein	Ratio	Protein	Ratio	Protein	Ratio	Protein	Ratio	Protein	Ratio	Protein	Ratio
Alb	1.75	Tmsb4x	1.7	Tfcp2	-2	Kdm5b	-2.5	F13a1	-1.43	Akt1	-1.43
Hpx	1.65	Oxnad1	1.65	Car8	-2	Hbb-b2	-2.5	Slc25a36	-1.43	Plbd2	-1.43
Heph	1.6	Smpd2	1.5	Rbm12b2	-1.67	Hbb-b1	-2	Dazap1	-1.43	Max	-1.43
Serpina3k	1.6	**Hbb-y**	1.48	Bgn	-1.67	Ctdp1	-1.818	Myg1	-1.43	Papola	-1.43
**Hbb-y***	1.56	Zswim8	1.45	**Krt73**	-1.67	Map3k7	-1.818	Syvn1	-1.43	Pvrl2	-1.43
Dpm3	1.55	Cldn11	1.45	Rpap3	-1.67	Exosc4	-1.818	Slc39a7	-1.43	Pla2g7	-1.43
Rpl24	1.55	Afp	1.44	**Dcun1d3**	-1.67	Wdr3	-1.67	Thtpa	-1.43	Paxbp1	-1.43
Inip	1.5	Vti1a	1.4	**Trip13**	-1.67	Uros	-1.67	Mtmr7	-1.43	**Trip13**	-1.43
Apoa1	1.5	Syt7	1.4	**Fam107b**	-1.67	Lrwd1	-1.67	Exosc2	-1.43	Ogfr	-1.43
Serpinc1	1.45	**Hist1h1c**	1.4	Homer3	-1.67	**Krt73**	-1.67	Kdm3b	-1.43	Nlgn4l	-1.43
Pbxip1	1.45	Fgd4	1.4	Cbln3	-1.64	Sap30	-1.67	Ybx3	-1.43	Chrm1	-1.43

Dnajb4	1.45	Fbxo2	1.4	**Krt4**	-1.56	**Hint2**	-1.67	Phf21a	-1.43	Smad2	-1.43
Col4a1	1.45	**Sulf1**	1.4	Rbmxl1	-1.54	Polb	-1.67	Ccdc102a	-1.43	**Lpp**	-1.43
Bai2	1.45	Etnk1	1.4	Exosc9	-1.54	Ezh2	-1.62	Ikbkg	-1.43	Rbbp4	-1.43
Rela	1.45	**Arpp21**	1.4	Sox1	-1.54	Tbck	-1.54	Rpap1	-1.43	Heatr5b	-1.43
**Hist1h1c**	1.45	Bai1	1.4	Mapk3	-1.54	Rwdd4a	-1.54	Efnb1	-1.43	**Ddr1**	-1.43
**Mrpl24**	1.45	**Mrpl24**	1.4	Vangl2	-1.54	**Fat4**	-1.54	Wipf2	-1.43	Ddrgk1	-1.43
Hbb-bh1	1.44	**Mc4-r**	1.4	Uap1l1	-1.54	**Fam107b**	-1.54	Hcn2	-1.43	**Dcun1d3**	-1.43
Usp19	1.4			**Ankrd52**	-1.54	Suz12	-1.54	Dhps	-1.43	Cand2	-1.43
Cacnb1	1.4			**Fat4**	-1.54	Panx1	-1.54	**Lpp**	-1.43	**Ncapd2**	-1.43
**Sulf1**	1.4			**Ints7**	-1.54	Gle1	-1.54	Lrsam1	-1.43	Mcam	-1.43
Ptrhd1	1.4			Ppp1r3f	-1.54	Mrc1	-1.54	Ephx1	-1.43	Cdkn2aipnl	-1.43
**Mc4-r**	1.4			Sqrdl	-1.54	Itih3	-1.54	Mrpl41	-1.43	Cdh4	-1.43
Tsr2	1.4			Hpcal1	-1.54	**Ints7**	-1.54	Pvalb	-1.43	Hexa	-1.43
**Arpp21**	1.4			Paxbp1	-1.54	Exosc1	-1.54	Irf2bpl	-1.43	**Bag1**	-1.43
				**Ncapd2**	-1.54	**Krt4**	-1.47	Ppp4r1	-1.43	Atrn	-1.43
				**Bag1**	-1.54	**Ubap2**	-1.43	**Hint2**	-1.43	Arnt2	-1.43
				**Ddr1**	-1.54	Tax1bp3	-1.43	**Ubap2**	-1.43	Apaf1	-1.43
				Polr2c	-1.54	Smarcd3	-1.43	Tgfbi	-1.43	Nt5dc3	-1.43
				Ncor2	-1.43	Sun2	-1.43	Ankrd46	-1.43	Azi1	-1.43
				Ttc27	-1.43	**Ankrd52**	-1.43			Lman2l	-1.43
				Ugdh	-1.43	Sephs1	-1.43			D2Wsu81e	-1.43

*. **Bold** type words show the proteins with the same trend of change in CCD and CRS F1 female mice.
